# Intramedullary Teratoma of Spine in an Adult Patient

**DOI:** 10.5334/jbsr.2835

**Published:** 2022-09-22

**Authors:** Suryansh Arora, Shishir Chumber, Kavita Vani

**Affiliations:** 1 RML, IN

**Keywords:** spinal teratoma, MRI, adult

## Abstract

Spinal teratomas are rare spinal tumors. Most of these present in children. We present the imaging findings of a spinal teratoma that was not symptomatic until adulthood.

**Teaching point:** Congenital spinal tumors may occasionally present for the first time in adulthood, and radiologists need to be familiar with the imaging findings.

## Introduction

Intramedullary tumors comprise a rare group of tumors accounting for 0.2–0.5% of spinal cord tumors [1]. A teratoma is a mitotic lesion that contains ectodermal, mesodermal, and endodermal elements. These tumors are very rarely found to present for the first time in adulthood. These are usually located in the thoracolumbar spine [2]. We present a case of an adult male with non-specific complaints diagnosed to be due to intramedullary teratoma of conus medullaris.

## Materials and Methods

A 46-year-old man presented with history of back pain for six months and gradual onset of numbness in bilateral lower extremities over the last two months. The patient was advised to undergo an MRI study of the lumbosacral spine with the clinical suspicion of degenerative disc disease. Magnetic resonance imaging (MRI) was carried out on 3T Siemens MAGNETOM scanner with phase array body coil.

## Results

As demonstrated in Figures A–E, imaging findings were consistent with the diagnosis of spinal intramedullary teratoma. No other congenital anomaly or features suggestive of degenerative pathology were identified. Radical surgical resection was carried out, and histopathology confirmed the diagnosis of teratoma.

**Figure:**
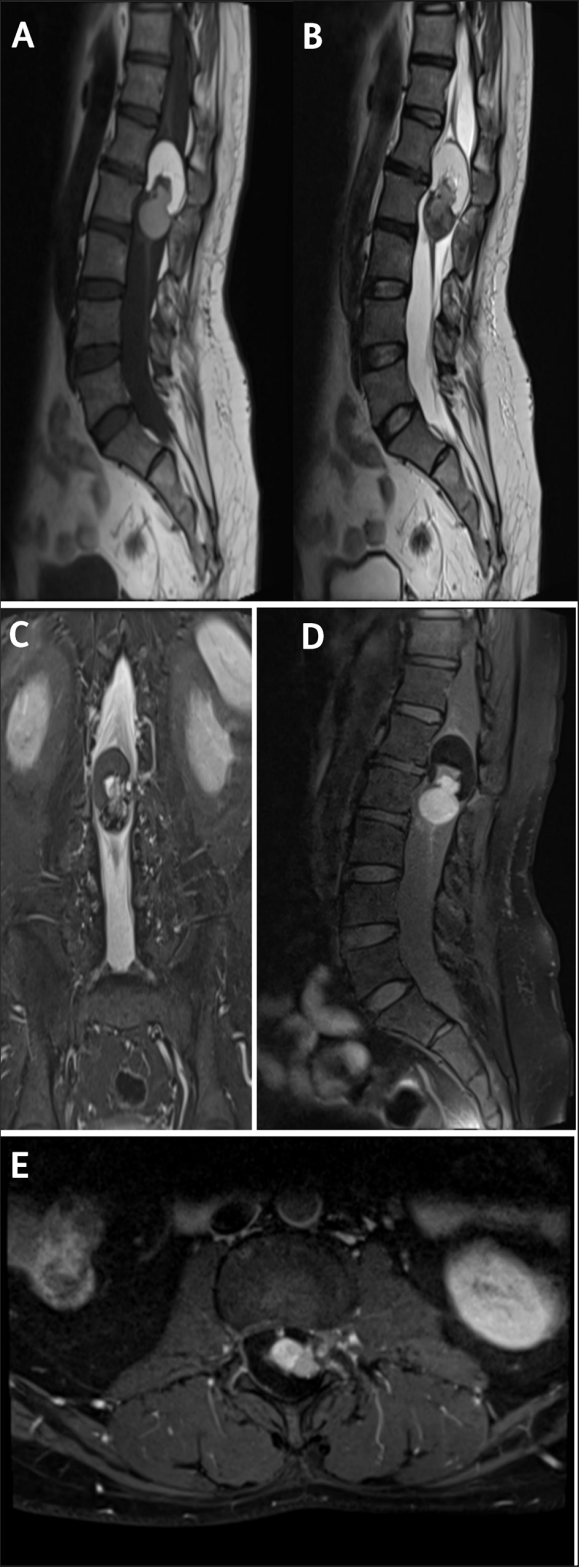
**(A)** Sagittal T1WI, **(B)** Sagittal T2WI, and **(C)** Coronal STIR image reveal an elongated multiloculated intramedullary lesion arising from conus medullaris, splaying cauda equina nerve roots with fat signal in cranial aspect of the lesion and heterogeneous hypointense signal on T2WI in caudal aspect of the lesion. Post-contrast T1WI FS image in **(D)** sagittal and **(E)** axial plane showing intense enhancement of caudal part of the lesion, suggestive of soft tissue component.

## Discussion

Conus medullaris is the most common location of spinal teratoma. Pathogenesis of these tumors is misplacement of multipotent germ cells during early embryonic life [3]. On MRI, spinal teratomas appear as well encapsulated lesions with both solid and cystic areas along with areas of fat signal and calcifications. They can be associated with various congenital anomalies, for example, spina bifida [4].

## Treatment

Ideal management of spinal teratomas is radical surgical excision. If located in highly functional areas, subtotal resection should be considered [4].

## Conclusion

Imaging with MRI is diagnostic in most cases of the rare tumor, spinal teratoma. A spinal teratoma clinically presenting for the first time in adulthood is extremely uncommon. The case presented herewith demonstrated classical features of a teratoma on MRI. These patients can improve significantly after surgical resection of the tumor. Hence radiologists need to be aware of the features of spinal teratoma.
